# Evaluation of flexural strength, impact strength, and surface microhardness of self-cured acrylic resin reinforced with silver-doped carbon nanotubes

**DOI:** 10.1186/s12903-024-03909-3

**Published:** 2024-01-31

**Authors:** Tamer M. Hamdy

**Affiliations:** Restorative and Dental Materials Department, Oral and Dental Research Institute, National Research Centre (NRC), Giza, Dokki, 12622 Egypt

**Keywords:** Polymethyl methacrylate (PMMA), Self-cure acrylic resin, Carbon nanotubes, Silver, Flexural strength, Flexural modulus, Impact strength, Microhardness

## Abstract

**Background:**

Poly-methyl methacrylate (PMMA) is a type of polymer mostly used to make denture bases. Self-cured acrylic resin (PMMA) can be used to repair a fractured acrylic denture base; however, even after repair, this area remains vulnerable. Carbon nanotubes (CNTs) could be used as a filler for polymer reinforcement. Furthermore, silver nanoparticles are efficient agents for the prevention of dental biofilm and improving their mechanical properties. The doping of CNTs with silver nanoparticles may lead to a synergistic interaction that is predicted to enhance the mechanical characteristics of the fillers.

**Objectives:**

The aim of the study was to assess the influnce of manual incorporation of 0.5% weight percent (%wt.) of silver doped carbon nanotubes (Ag-doped CNTs) into commercial self-cured PMMA on its flexural strength, impact strength, and surface microhardness.

**Methods:**

In this investigation, a total of 60 specimens comprised of acrylic resin were employed. They are divided into two main groups: (a) the control group, which was made by using liquid monomer and commercial self-cured PMMA powder; and (b) the modified group, prepared by hand mixing the purchased silver-doped CNTs powder (0.5% wt.) to self-cured PMMA powder (99.5%wt.), and then the blended powder was incorporated into the liquid monomer. Flexural strength, flexural modulus, impact strength, and surface microhardness were evaluated. Independent sample t-tests were used to statistically analyze the data and compare the mean values of flexural strength, flexural modulus, impact strength, and surface microhardness (*p-value* ≤ 0.05).

**Results:**

The flexural strength of the modified groups with Ag-doped CNTs (132.4 MPa) was significantly greater than that of the unmodified (control) groups (63.2 MPa). Moreover, the flexural modulus of the modified groups with Ag-doped CNTs (3.067 GPa) was significantly greater than that of the control groups (1.47 GPa). Furthermore, the impact strength of the modified groups with Ag-doped CNTs (11.2 kJ/mm^2^) was significantly greater than that of the control groups (2.3 kJ/mm^2^). Furthermore, the microhardness of the modified groups with Ag-doped CNTs (29.7 VHN) was significantly greater than that of the control groups (16.4 VHN), (*p-value = 0.0001*).

**Conclusion:**

The incorporation of 0.5% wt. silver doped CNTs fillers to the self-cured acrylic resin enhanced its flexural strength, flexural modulus, impact strength, and surface microhardness.

## Background

Most dental applications depend on polymers [1–4]. One of the most widely used materials in the manufacturing of removable denture bases is acrylic resin [5]. Poly-methyl methacrylate (PMMA) is the main component of heat- or self-cured acrylic resins. Heat-cured acrylic resin is primarily used in the manufacturing of denture bases. Self-cured acrylic resin is used to repair dentures in order to prevent warping and destroying the denture’s broken parts [6, 7].

Fracture of acrylic dentures is an issue commonly encountered by denture wearer patients, and it occurs as a result of insufficiency in material characteristics, technical design, and stresses to which dentures are subjected during service [8]. The most frequent causes of the fracture are improper placement and occlusion of the teeth for the upper denture, as well as accidentally dropping the denture in the case of the lower one and its imperfect fit and stability [9].

High flexural stresses that occur during mastication when using dentures might cause fractures of denture bases inside the mouth [10]. However, fractures outside of the mouth typically occur accidentally, like when a denture is dropped [11]. A Denture rebase or repair of the fractured parts by self-cured acrylic resins is thought to be an excellent strategy, conserving time and expenses. When choosing to build an entirely new denture [12].

All acrylic-based dentures are exposed to internal or external oral stresses [13]. If a denture is suddenly dropped, it could fracture due to its low impact resistance [14]. However, fracture of the denture may occur as a result of increased flexural stresses due to excessive biting force, improper design, an inappropriate occlusal plane, unbalanced occlusion, and a poorly fitted denture [15, 16]. Microhardness is an intrinsic surface property of a material, denoting its resistance to scratching or indentation [17]. Some reports claimed that there is direct correlation between microhardness and wear resistance [18], which is mainly due to incorporation of nano-sized filler particles to the organic matrix, providing a composite with more resistance to wear and scratching [19–21]. Despite the desired properties of PMMA, there is an ongoing demand to enhance the mechanical properties of the acrylic denture base [22, 23].

One of the most commonly used materials for denture repair is self-cured acrylic resin. Because self-cured acrylic resins have lower mechanical properties than heat-cured acrylic resins, it is possible to anticipate recurrent fractures. A concern about the improvement of the mechanical properties of the denture base repair materials should be expressed [24]. There have been numerous attempts to incorporate different types of fillers into PMMA-based polymers to reinforce them, such as rubbers, fibers, ceramics, and metals [22, 25–29]. Recent studies proved that addition of short E-glass fiber improved the flexural strength of self-cured PMMA materials [30]. Moreover, other study showed that both zirconia and boron nitride fillers could be enhancing the mechanical properties of self-cured PMMA materials [31].

Carbon nanotubes (CNTs) are one of the nanomaterials which promise to accelerate a revolution in the disciplines of dentistry due to their tremendous potential for biological applications and enhanced mechanical and physical properties [32–35]. CNTs are mostly used in tissue engineering, either as promoting agents to repair damaged tissues or as scaffolds to create a structural integrity that is favorable for the incorporation of cells [36]. CNTs could be used as a fillers for polymers reinforcement [37]. Multi-walled CNTs could be utilized as superior fillers due to their higher surface area and increased loading capacity [38]. Accordingly, their enhanced mechanical characteristics suggest that they would be employed as an emerging polymer filler [34, 39]. According to studies, functionalization of CNT is the most appropriate to create a carbon-based polymeric material to enhances the mechanical characteristics of the substrates [36].

Silver (Ag) nanoparticles have long been utilized in dentistry due to their strengthening effect, in addition to their antimicrobial activity [40]. Combining CNTs with other nano-metallic particles may be able to provide nanofillers with enhanced properties [41]. The combination of CNTs with Ag nanoparticles may lead to a synergistic interaction to enhance the mechanical properties of the fillers [38]. Thus, the aim of this study was to assess the influence of manual addition of 0.5% weight percent (%wt.) of silver-doped CNT nanoparticles into self-cured PMMA on its flexural strength, impact strength, and surface microhardness. The concentration of 0.5% wt. of Ag-doped CNTs was chosen as it represents the maximum amount by trial that could be added without a visually obvious change in the color of the specimens. The null hypothesis stated that the incorporation of 0.5%wt. silver doped CNTs to chemically-cured PMMA would have no affect the flexural strength, impact strength, or surface microhardness compared to the non-treatment group (control).

## Methods

The present experimental study was approved by the Medical Research Ethical Committee (MREC) of the National Research Centre (NRC), Cairo, Egypt (Reference number: 440542023). The sample size was calculated using the G*Power (version 3.1.9.7) sample size calculator based on means and standard deviations [42, 43]. The estimated sample size was 10 per group.

In this study, a commercially available conventional self-cured acrylic resin was utilized (Acrostone Cold Cure Acrylic Resin, Acrostone Co., England). Commercial multi-walled CNT doped with 12%wt. nano-silver powder was also employed (Nanografi Nano Technology, Jena, Germany). The Ag-doped CNT has the following features according to the manufacture’s specifications [44]: average particle length between 15 and 25 nm, average inside diameter of 5 nm, outside diameter less than 50 nm, and an average purity of over 97%wt.

Specimens were divided into two main groups: (a) the control group was prepared by mixing the commercial self-cured PMMA powder to its liquid monomer in a ratio of 3:1 by volume according to the manufacturer’s instructions; (b) the modified group was prepared by manually adding 0.5% of the purchased Ag-doped CNT nanoparticles to self-cured PMMA powder (99.5%) (by weight). Hand-mixing was done by blending the two powders in a rotational motion for 5 minutes using a spatula and then shaking in a closed container for an additional 5 minutes. Then, the blended powder was incorporated into the liquid monomer in a ratio of 3:1 by volume, as previously described.

When the mixed acrylic resin had reached the dough stage, it was packed in certain molds according to each test type. The produced specimens were removed from the molds after 10 minutes to ensure adequate polymerization process as recommended by the manufacture. The specimens were visually examined to ensure that the surface was smooth and flat and showed no faults, voids, or porosity; if not, they were discarded. The specimens were then placed in distilled water at 37 °C for 24 hours before testing [29, 45]. The parameters measured in this study were flexural strength, impact strength, and surface microhardness. For each test, 20 specimens were prepared from each group (control and modified).

### Flexural properties testing

A flexural strength test was evaluated using 3-point bending according to ISO 20795-1 [46]. Using a metallic mold, specimens of 64 mm (length) × 10 mm (width) × 3.3 mm (thickness) were prepared [46]. The specimens were examined by a universal testing machine (Model 3345; Instron Industrial Products, Norwood, MA, USA). The load was applied to the center of the specimens, which were kept over a 2-point support span of 50 mm apart with a crosshead speed of 5 mm/min, with a load cell of 500 N. The specimens were loaded till fracture. The load at fracture was represented in Newtons (N). The metallic mold was placed in water at a temperature of 50 °C for 5 minutes to confirm a complete polymerization reaction. The specimens were then kept for 50 ± 2 hours at 37 ± 1 °C in an incubator with distilled water before the flexural strength test.

The flexural strength (FS) was calculated in (MPa) with the following equation [29, 47]: FS = 3PL/2bh^2^. Where; (P) is the maximum load at fracture (N); (L) is the distance between the supports (mm); (b) is the width (mm); (h) is the height of the specimen (mm).

The flexural modulus (E) was determined in (GPa) using the following formula: E *=* PL^3^/4bh^3^d. Where; (P) is the maximum load at fracture (N); (L) is the distance between the supports (mm); (b) is the width (mm); (h) is the height of the specimen (mm); (d) is the deflection corresponding to the load (mm).

### Impact strength test

The impact strength was examined by a Charpy tester (Ceast-Resil impactor, Type 6,967,000, Torino, Italy), following ISO standard 179–1:2010 [48]. At the center of each specimen, a V-shape notch was made. Each specimen was clamped horizontally from both ends, and a swinging pendulum was utilized to hit the specimen in the center to induce fracture. The energy absorption and impact energy (in joules) were assessed using scale readings on fractured specimens. Charpy impact strength was calculated using the following formula [49]: Impact strength (kJ/m^2^) = E/TW, where: E = the absorbed energy (kJ), W = the specimen width (m), T = the specimen thickness at the notch base (m).

### Surface microhardness test

**Fig. 1:**
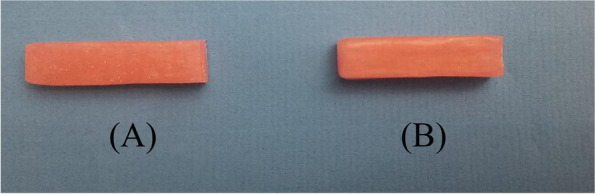
Representatively prepared specimens of the surface microhardness test (**a**) control specimen (**b**) modified specimen

Surface microhardness was investigated by a digital Vickers hardness tester (NEXUS 400TM, INNOVATEST, model no. 4503, The Netherlands). Using a stainless-steel mold, specimens with the dimensions (65 mm × 10 mm × 2.5 mm) were created as shown in Fig. 1 [50, 51]. The indentations were made within 20 seconds of loading 500 g at 20 magnification [50, 51]. The Vickers microhardness number (VHN) value was calculated automatically using the formula: VHN = 1.8544 P/d^2^, where (p) is the applied force in kilograms and (d) is the mean of the two diagonals gained from the indentation in mm.

## Statistical analysis

The statistical analysis was performed using the Statistical Package for the Social Sciences (12.0, SPSS Inc., IBM, USA). According to the results of the normality test conducted with the Shapiro-Wilk and Kolmogorov-Smirnov tests, independent sample *t*-tests were used to statistically to compare mean values of flexural strength, flexural modulus, impact strength, and microhardness between the two groups (control PMMA and modified PMMA with Ag-doped CNTs). The significance level was set at *p-value* ≤ 0.05.

## Results

The mean and standard deviation values for flexural strength (MPa), flexural modulus (GPa), impact strength (kJ/mm^2^), and Vickers microhardness number (VHN) for the chemical-cured PMMA (control group) and the modified group with Ag-doped CNTs are represented in Table 1. The flexural strength of the modified groups with Ag-doped CNTs (132.4 MPa) was significantly greater than that of the control groups (63.2 MPa). Moreover, the flexural modulus of the modified groups with Ag-doped CNTs (3.067 GPa) was significantly greater than that of the control groups (1.47 GPa). Furthermore, the impact strength of the modified groups with Ag-doped CNTs (11.2 kJ/mm^2^) was significantly greater than that of the control groups (2.3 kJ/mm^2^). Furthermore, the microhardness of the modified groups with Ag-doped CNTs (29.7 VHN) was significantly greater than that of the control groups (16.4 VHN), *p-value* ≤ 0.05.

**Table 1:** Results of performed tests for the chemical-cured PMMA, unmodified (control) groups and modified groups with Ag-doped CNTs

Test	Self-cured PMMA (control groups)	Modified groups with Ag-doped CNTs	*p-value* (Sig.)
Flexural strength (MPa)	63.2 ± 0.3	132.4 ± 0.2	*p* = 0.0001*
Flexural modulus (GPa)	1.47 ± 0.4	3.067 ± 0.1	*p* = 0.0001*
Impact strength (kJ/mm^2^)	2.3 ± 0.2	11.2 ± 0.1	*p* = 0.0001*
Microhardness (VHN)	16.4 ± 0.6	29.7 ± 0.5	*p* = 0.0001*

*Corresponds to statistically significant difference (*p-value* ≤ 0.05)

## Discussion

Fracture resistance for acrylic resins is a key concern due to the high costs associated with maintaining acrylic prosthetics [52, 53]. The primary material used to make denture bases is still PMMA acrylic resin [5, 54]. Although the advantages of PMMA self-cured acrylic resin as a denture base repair material are many, fractures are very commonly encountered under flexural and/or impact stresses. Thus, there is a significant need to improve the mechanical characteristics of conventional PMMA-based, self-cured denture bases [14, 30]. Repeated fracture of the repaired denture base often occurs within the repaired, self-cured resin [55].

Researchers have made numerous attempts to improve the mechanical performance of the self-cured PMMA resin [22, 54, 56, 57]. The improvement of the mechanical properties of acrylic resin could be achieved through reinforcement with several fibers and nanoparticles [52, 54].

The international standard ISO for dentistry base polymers states, the acrylic resin should exhibit a minimum standard limit for flexural strength of no less than 60 MPa [46]. The clinical service longevity of the denture is improved with increased flexural strength [58]. On the other hand, the abrupt application of force to dentures might cause them to fracture due to impact stresses. Such fractures are more likely to occur as a result of patients accidentally dropping their dentures while cleaning them [59]. Additionally, the lower values of surface microhardness of chemical-cured acrylic denture bases makes them more prone to being scratched, weakening the denture base, and encouraging the collection of debris [60]. The superior mechanical properties of the CNTs open the door to using them in dental materials to provide a novel functional application [61].

Ag-nanoparticles could be added in combination with CNTs to produce innovative fillers that will be able to improve the mechanical and surface characteristics of conventional PMMA-based self-cured acrylic resin [38, 39, 62]. The percentage of addition of 0.5%wt. of Ag-doped CNTs into commercially chemical-cured PMMA was chosen as previous studies proved that the addition of 0.5%wt. of multi-walled CNTs into PMMA provided minimum polymerization shrinkage, [37] and maximum tensile strength [63].

The purpose of this study was to evaluate and compare the influences of adding 0.5%wt. of Ag-doped CNTs to commercially self-cured PMMA by manual mixing on the flexural strength, impact strength, and surface microhardness to improve some mechanical properties of the denture base.

The null hypothesis was rejected, indicating that the flexural strength, flexural modulus, impact strength, and surface microhardness of the reinforced chemical-cured PMMA acrylic resin were all significantly affected by the addition of 0.5%wt. Ag-doped CNTs to commercial chemical-cured PMMA.

From previous studies, it was concluded that using of fillers with a small average particle size and narrow size range can obtain an improved strengthening effect on the composites [64, 65]. Furthermore, it has been noted that a relatively small weight percentage of nano-sized particle can result in a significantly improved mechanical properties [66].

The findings of this investigation showed that the addition of 0.5%wt. Ag-doped CNTs significantly improved the flexural strength and flexural modulus of chemical-cured acrylic resin, which may be attributed to the restriction of the slippage movement between the polymer segments in relation to each other by the effect of CNTs [67–69]. Moreover, the distribution of nano-scaled particles within the conventional polymer exhibits an enormous surface area, which interfaces for stress transfer [5]. The results of the current study agreed with other studies conducted to improve the flexural strength of the self-cured acrylic resin by incorporation of either E-glass fiber or titanium oxide [22, 30].

The increase in impact strength values in the modified groups with Ag-doped CNTs could be obtained due to the effect of the inclusion of strong and stable Ag-doped CNT fillers into polymer. These may be attributed to the arrangement of carbon nanotubes in a hexagonal ring, which reduce the segmental motion [70]. Thus, increasing the impact strength by obstruction of the crack propagation [71]. The current study’s findings were consistent with earlier research aimed at enhancing the self-cured acrylic resin’s impact strength through reinforcing using zirconia and boron nitride nanofillers [31].

The modified groups with Ag-doped CNT nanoparticles showed increased surface microhardness values, which could be explained by the existence of a homogenous distribution of hard nanoparticle fillers of Ag-doped CNTs within the acrylic denture base materials [72–76]. These results confirm the results obtained from other study conducted to investigate the surface microhardness the conventional glass ionomer reinforced with Ag-doped CNTs [35].

The results of this study are consistent with prior studies that showed the modification of light-cured PMMA, heat-cured PMMA, and bone cement PMMA by the inclusion of CNTs nanoparticles exhibits an improvement in their mechanical properties compared to conventional ones [5, 37, 63, 69]. Finally, this in vitro study represents a primary phase to investigate the capability of the functionalized Ag-doped CNT nanoparticle to improve the mechanical properties of the conventional PMMA-based self-cured acrylic resin.

The limitations of the current study include the restriction of the mechanical investigation on the flexural strength, impact strength, and surface microhardness only; therefore, it is recommended to investigate other mechanical properties such as compressive strength and fatigue limit. Furthermore, the experimental conditions did not perfectly fit the clinical situations.

It was highly recommended to do further investigation about the bond strength at interface as it represents the most common site for failure. Moreover, it beneficial to perform further studies to perform a morphological and chemical characterization of the modified PMMA groups with Ag-doped CNTs. Further investigations about surface roughness are recommended to assess any possible determinantal effect of the added fillers.

## Conclusions

The reinforcement of self-cured PMMA acrylic resin by the addition of 0.5% wt. silver-doped CNT fillers provide promising outcomes on flexural strength, flexural modulus, impact strength, and surface microhardness.

## Data Availability

The datasets generated during and/or analyzed during the current study are not publicly available due to institutional policy but are available from the corresponding author on reasonable request.

## Abbreviations

PMMA: Poly-methyl methacrylate
CNTs: carbon nanotubes
% wt: weight percent
MREC: Medical Research Ethical Committee
NRC: National Research Centre
ISO: International Organization for Standardization
N: Newtons
fs: flexural strength
VHN: Vickers microhardness number.
